# Supplementary material for: How Carvedilol activates β2-adrenoceptors
Source: Nat Commun. 2022 Nov 19;13:7109. doi: 10.1038/s41467-022-34765-w (PMC9675828; doi:10.1038/s41467-022-34765-w)
Supplement: Supplementary file 1 — Supplementary Information [file 41467_2022_34765_MOESM1_ESM.pdf]

Supplementary Information

How Carvedilol activates β_2-adrenoceptors

Tobias Benkel[1,11], Mirjam Zimmermann[2], Julian Zeiner[1], Sergi Bravo[1], Nicole Merten[1], Victor Jun Yu Lim[3], Edda Sofie Fabienne Matthees[4], Julia Drube[4], Elke Miess-Tanneberg[5], Daniela Malan[6], Martyna Szpakowska[7], Stefania Monteleone[3], Jak Grimes[8], Zsombor Koszegi,[8] Yann Lanoiselée[8], Shannon O'Brien[8], Nikoleta Pavlaki[9], Nadine Dobberstein[2], Asuka Inoue[10], Viacheslav Nikolaev[9], Davide Calebiro[8], Andy Chevigné[7], Philipp Sasse[6], Stefan Schulz[5,12], Carsten Hoffmann[4], Peter Kolb[3], Maria Waldhoer[2,13], Katharina Simon[1], Jesus Gomeza[1], and Evi Kostenis[1]

[1] Molecular, Cellular and Pharmacobiology Section, Institute of Pharmaceutical Biology, University of Bonn, 53115 Bonn, Germany.

[2] InterAx Biotech AG, 5234 Villigen, Switzerland.

[3] Department of Pharmaceutical Chemistry, Philipps-University of Marburg, 35032 Marburg, Germany.

[4] Institute for Molecular Cell Biology, CMB-Center for Molecular Biomedicine, Jena University Hospital, Friedrich Schiller University of Jena, 07745 Jena, Germany.

[5] Institute of Pharmacology and Toxicology, Jena University Hospital, Friedrich-Schiller-University of Jena, 07747 Jena, Germany.

[6] Institute of Physiology I, Medical Faculty, University of Bonn, 53115 Bonn, Germany.

[7] Department of Infection and Immunity, Luxembourg Institute of Health (LIH), L-4354 Esch-sur-Alzette, Luxembourg.

[8] Institute of Metabolism and Systems Research and Centre of Membrane Proteins and Receptors (COMPARE), University of Birmingham, Birmingham B15 2TT, UK.

[9] Institute of Experimental Cardiovascular Research, University Medical Center Hamburg-Eppendorf, 20246 Hamburg, Germany.

[10] Graduate School of Pharmaceutical Science, Tohoku University, Sendai 980-8578, Japan.

[11] Research Training Group 1873, University of Bonn, 53127 Bonn, Germany.

[12] 7TM Antibodies GmbH, 07745 Jena, Germany

[13] Present address: Ikherma Consulting Ltd, Hitchin SG4 0TY, UK

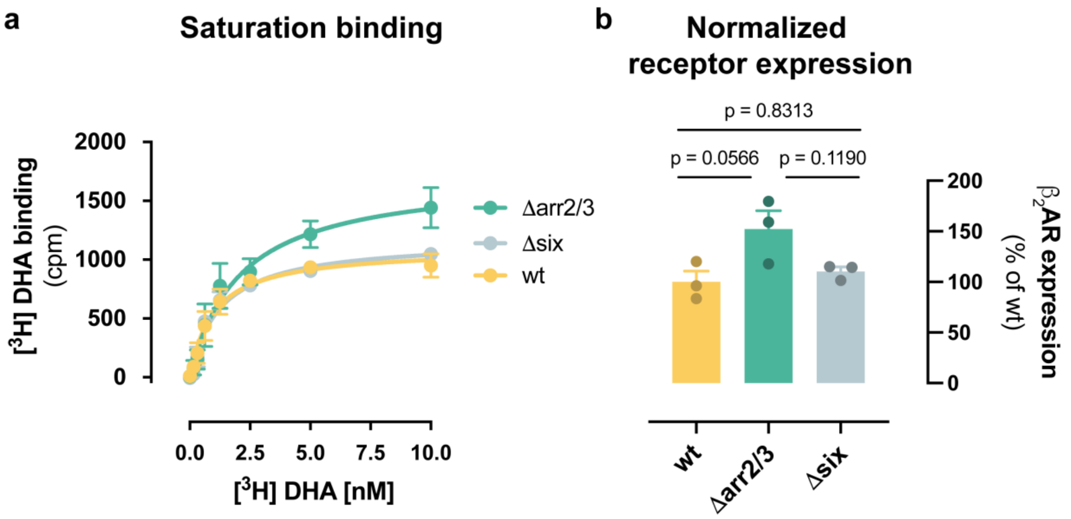

Supplementary Fig. 1: β_2ARs are expressed at comparable abundance across all HEK293 cell lines. [^{3}H]-DHA saturation binding experiments on membranes collected from wt and genome-edited HEK293 cells stably expressing SNAP-β_2ARs. **a** Summarized saturation binding isotherms of membranes treated with increasing concentrations of [^{3}H]-DHA. The data was fitted to a one site specific binding model using GraphPad Prism 9. **b** B_{max} values derived from the data in **(a)** and normalized to SNAP-β_2AR wt HEK293 cells. Data is shown as mean +/± SEM of three independent experiments, each performed in duplicate determinations. Data was analysed by one-way ANOVA with Tukey's post hoc test. Source data are provided as a Source Data file.

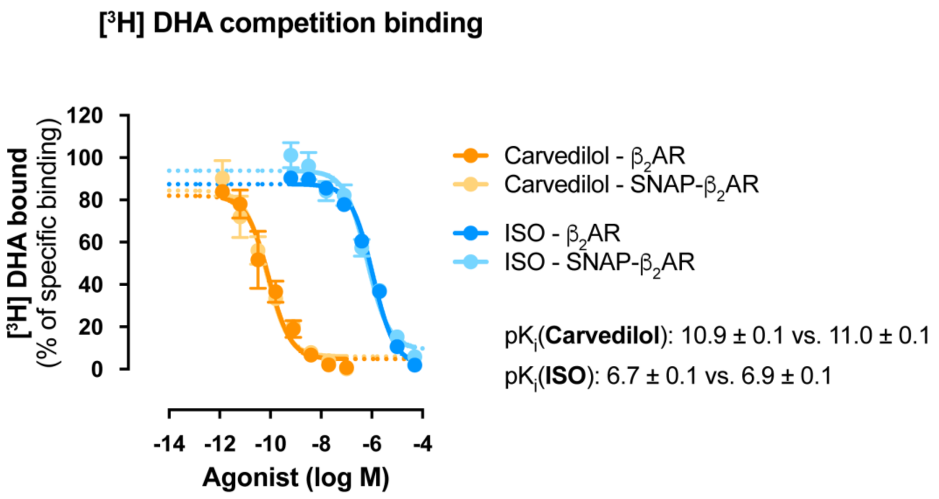

Supplementary Fig. 2: The addition of a SNAP-tag does not impair ligand binding to the β_2AR. Heterologous radioligand competition binding experiments with [^{3}H]DHA on membranes collected from wt HEK293 cells after transient transfection with β_2ARs or SNAP-β_2ARs. Transfected cells were incubated with 1 nM [^{3}H]DHA alongside increasing concentrations of carvedilol or ISO. Nonspecific binding was determined in the presence of 1 μM ICI-118,551. Specific binding, in the absence of agonist, was set to 100 %. pK_i values were calculated by fitting the data to a one site-specific binding model using GraphPad Prism 9. Data is shown as mean ± SEM of three biological replicates performed in technical duplicate. Source data are provided as a Source Data file.

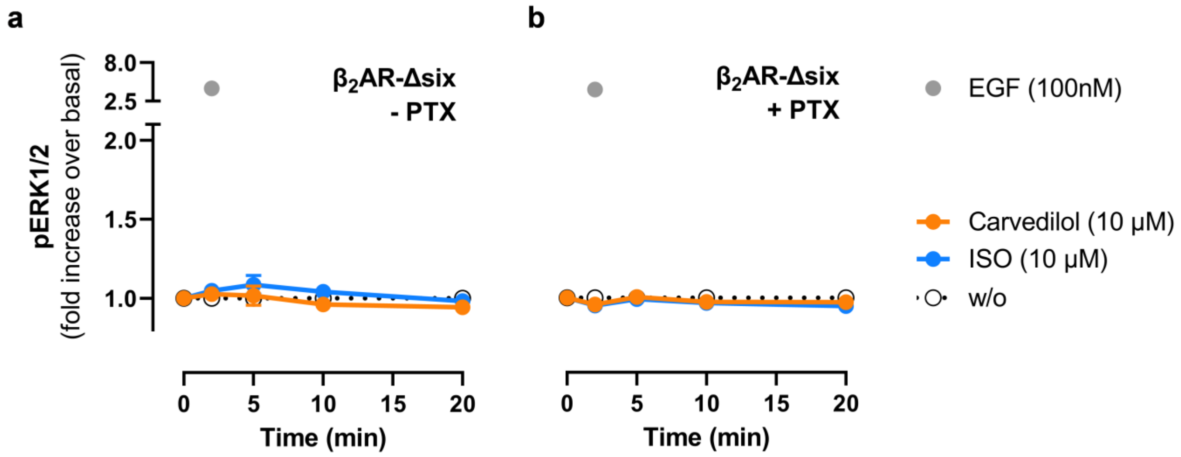

Supplementary Fig. 3: Ligand-mediated β_2AR ERK1/2 phosphorylation is undetectable in the presence and absence of active Gi/o proteins. Temporal pattern of ERK1/2 phosphorylation in Δsix cells stably expressing SNAP-β_2ARs upon stimulation with either carvedilol or ISO in the absence **(a)** or presence **(b)** of the $G\alpha_{i/o}$ inhibitor PTX. Data is depicted as mean ± SEM of three independent biological replicates. w/o, without. Source data are provided as a Source Data file.

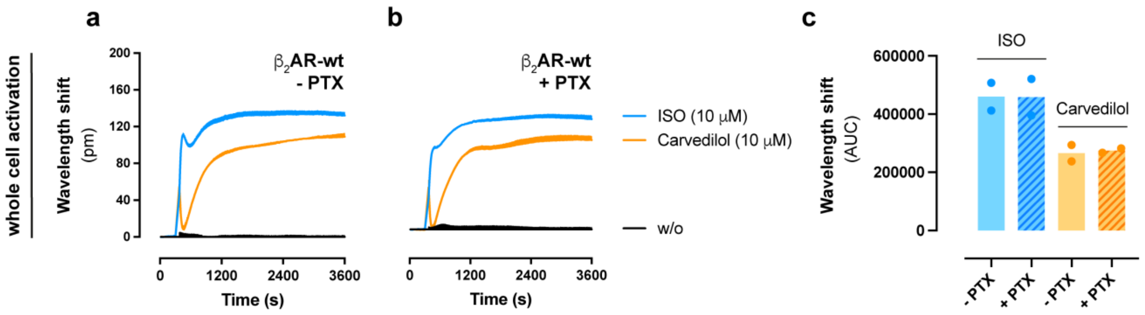

Supplementary Fig. 4: Whole cell responses triggered by ligand-stimulated β_2AR do not require endogenous G$\alpha_{i/o}$ activation. a, b Representative optical recordings of cell morphological changes in wt HEK293 cells stably expressing SNAP-β_2ARs upon stimulation with either carvedilol or ISO, in the absence **(a)** or presence **(b)** of the G$\alpha_{i/o}$ inhibitor PTX. **c** Corresponding area under the curve (AUC) values. Representative kinetic traces **(a, b)** are mean + SD of a representative experiment, measured in triplicate. Summarized data **(c)** is depicted as mean of two independent experiments, measured in triplicate. pm, picometer; w/o, without. Source data are provided as a Source Data file.

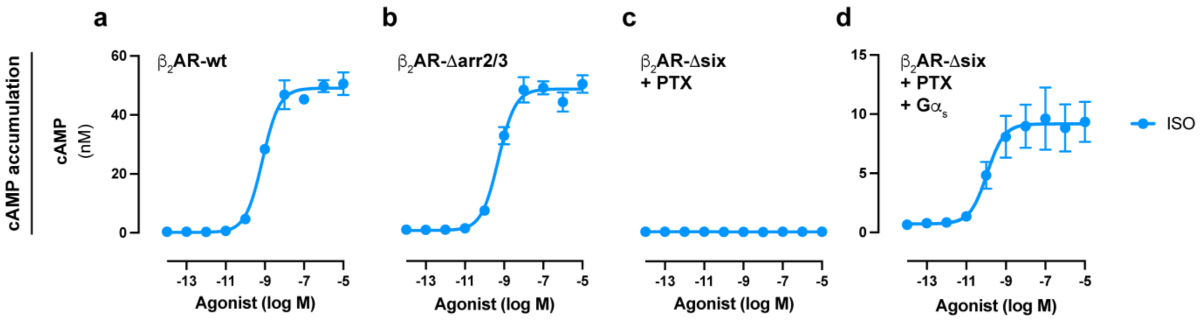

Supplementary Fig. 5: Continuous Y-axis view of Fig. 1i-l showing the ISO-mediated cAMP accumulation.

Summarized data are presented as means ± SEM (n = 3). Source data are provided as a Source Data file.

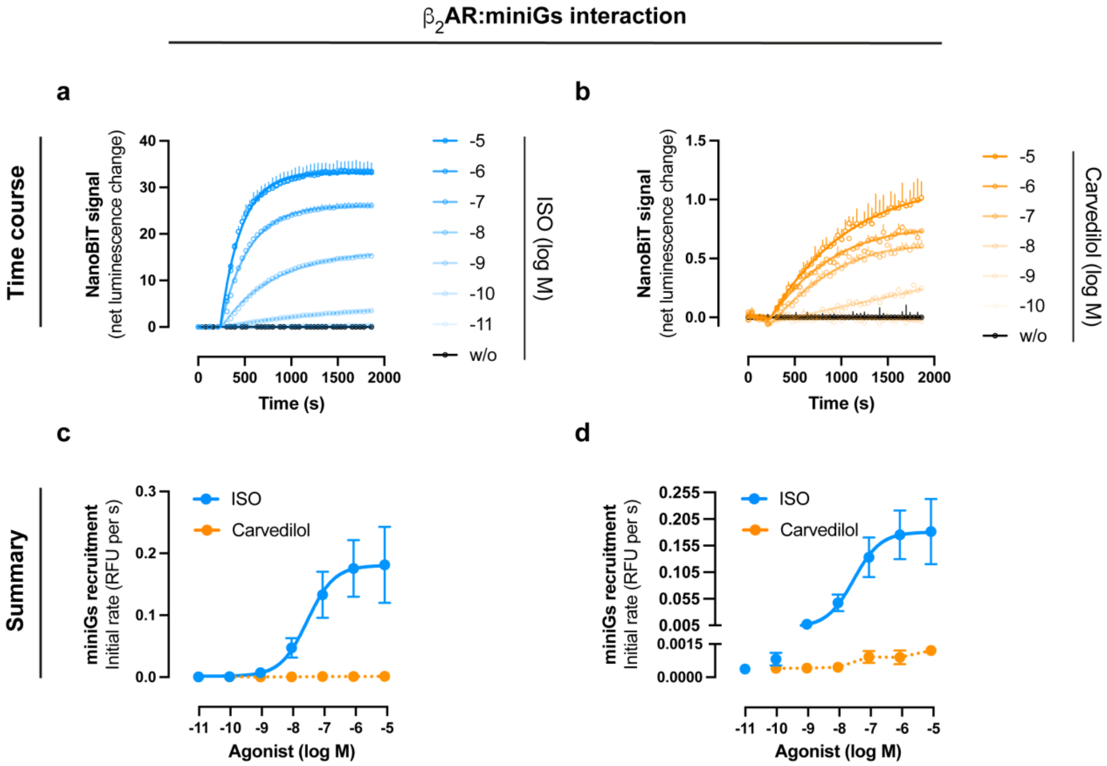

Supplementary Fig. 6: Carvedilol-induced slow onset low efficacy recruitment of miniGs to the β$_2$AR. a, b Representative kinetic traces of NanoBiT complementation in wt HEK293 cells transiently transfected with β$_2$AR-SmBiT and LgBiT-miniGs after treatment with increasing concentrations of either ISO **(a)** or carvedilol **(b)**. **c, d** Corresponding concentration–response analysis of the initial rate of β$_2$AR:miniGs interaction for ISO and carvedilol depicted with continuous **(c)** and split-axis view **(d)**. Kinetic traces are shown as mean + SD of a representative experiment, measured in duplicate. Summarized data is depicted as mean ± SEM of three independent experiments. w/o, without. Source data are provided as a Source Data file.

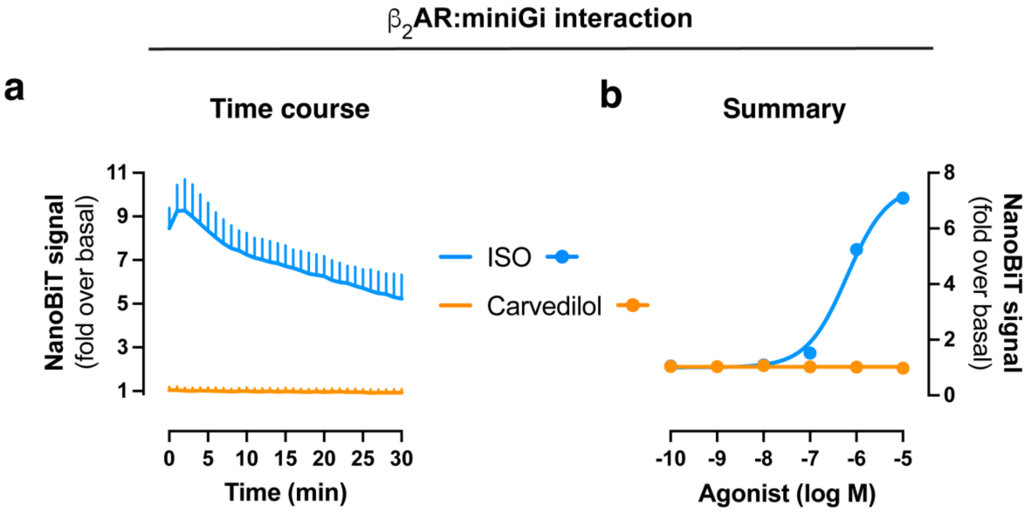

Supplementary Fig. 7: Carvedilol does not measurably recruit miniGi to the β_2AR. **a** Summarized kinetic traces of NanoBiT complementation in wt HEK293 cells transiently transfected with β_2AR-SmBiT and LgBiT-miniGi after treatment with either 10 µM carvedilol or 10 µM ISO. **b** Concentration-effect curves derived from the luminescence signal traces of the respective ligand concentrations. Kinetic traces are means + SD of five independent experiments, measured in duplicate, and their derived concentration-effect data are shown as mean ± SEM. Source data are provided as a Source Data file.

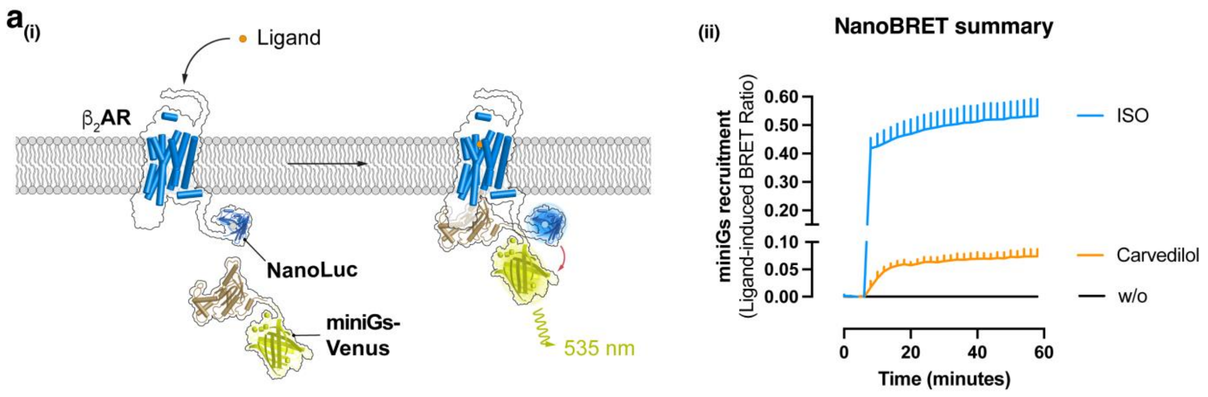

Supplementary Fig. 8: Measurement of miniGs recruitment to the ligand-activated β_2AR. (a$_i$) Assay principle of NanoBRET-based interaction between miniGs and the ligand-activated β_2AR, utilizing a β_2AR C-terminally tagged with NanoLuc® and miniGs N-terminally fused to the yellow fluorescent protein Venus. Recruitment of miniGs to the receptor allows resonance energy transfer between NanoLuc® and Venus. **(a$_{ii}$)** Time course of BRET changes provoked by the treatment with either 10 µM carvedilol or 10 µM ISO in wt HEK293 cells transiently transfected with the labeled constructs. Kinetic data is shown as mean + SD of three independent biological replicates. w/o, without. Source data are provided as a Source Data file.

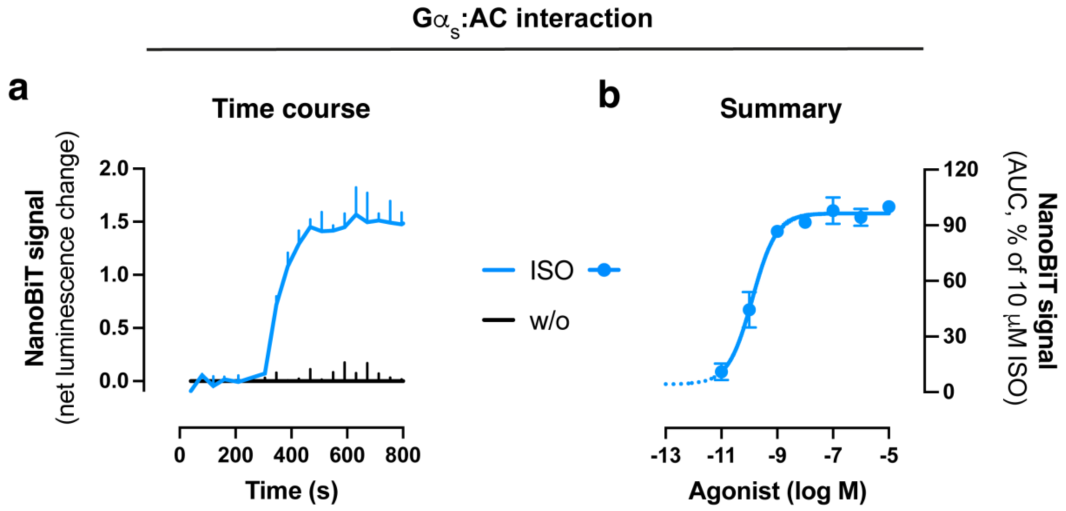

Supplementary Fig. 9: Continuous Y-axis view of Fig. 2c showing the ISO-mediated interaction between Gα$_s$ and AC5.
a Kinetic traces of a representative experiment are shown as means + SD of 2 technical replicates. **b** Summarized data are presented as means ± SEM (n = 3). Source data are provided as a Source Data file.

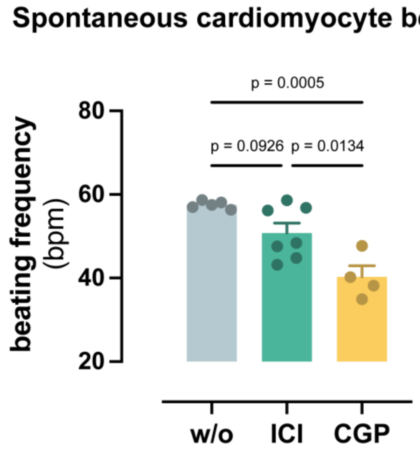

Supplementary Fig. 10: β-adrenergic control of spontaneous cardiomyocyte beating. Contribution of β_1ARs and β_2ARs to the spontaneous beating frequency of neonatal mouse ventricular myocytes (NMVM). Shown is the effect of solvent (w/o, n = 5), 10 µM β_2 antagonist ICI118.551 (ICI, n = 7) or 10 µM β_1 antagonist CGP20712A (CGP, n = 4) on spontaneous cardiomyocyte beating, as detected by impedance recordings. Each data point represents an individual well from three different animal preparations. Data is shown as mean + SEM and was analysed by one-way ANOVA with Tukey's post hoc test. bpm, beats per minute. Source data are provided as a Source Data file.

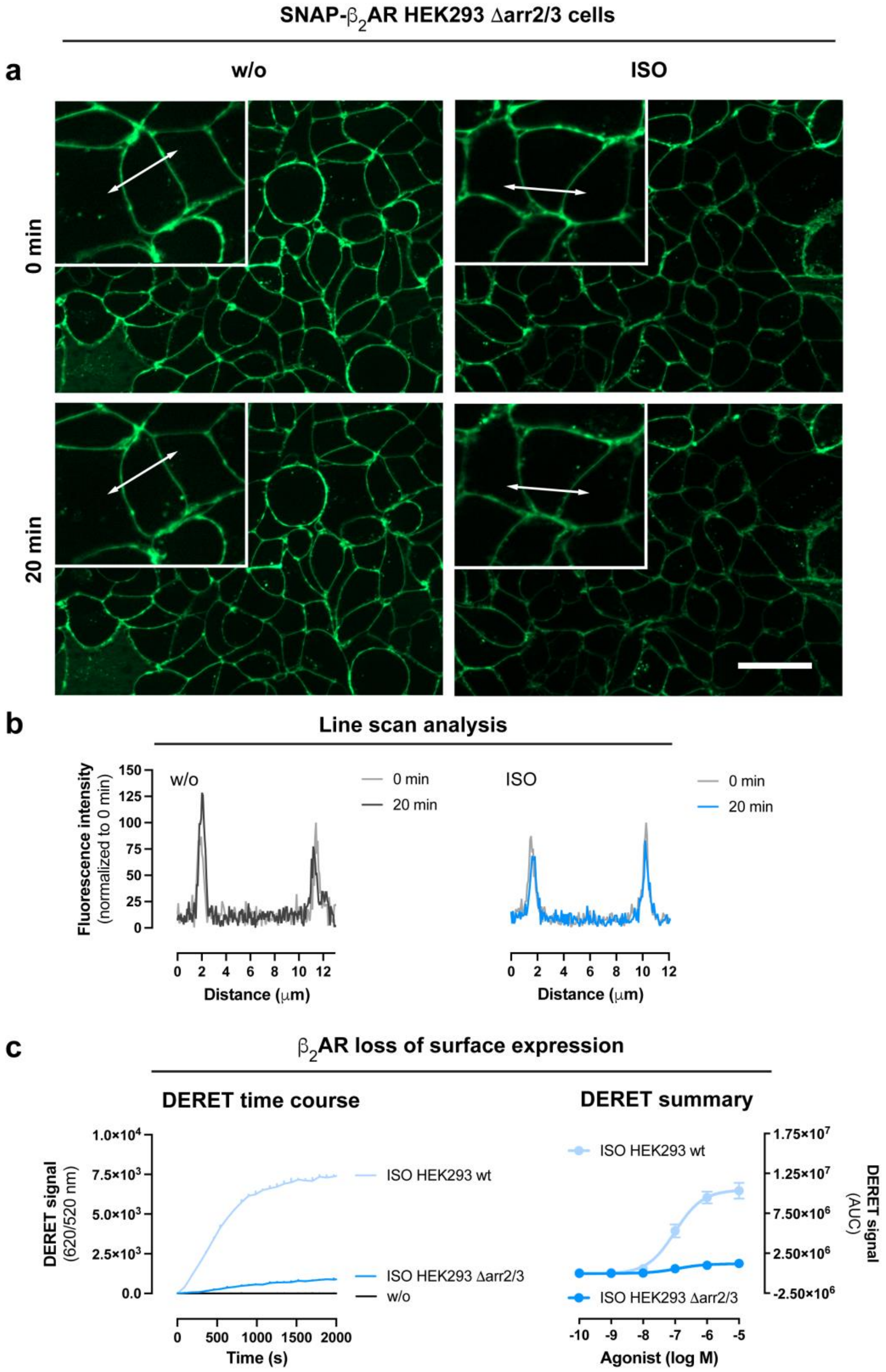

Supplementary Fig. 11: β_2AR internalization in HEK293 cells is an arrestin-dependent process. ISO-mediated internalization in SNAP-β_2AR HEK293 Δarr2/3 cells. **a** Structured-illumination micrographs (representative of three independent experiments) of HEK293 Δarr2/3 cells stably expressing SNAP-β_2ARs treated with buffer (w/o) or 10 μM ISO for 20 minutes using focus control. **b** Line scan analysis along a trajectory, indicated by double-faced arrows, of buffer- or ISO-mediated changes in fluorescence intensity. **c** Kinetic recording of ligand-induced DERET (diffusion enhanced resonance energy transfer, a measure for β_2AR surface loss; see DERET assay principle in Fig. 4c) and corresponding concentration-response curves. For comparability reasons, ISO-mediated DERET signals in the presence of arrestins (cf. Fig 4d) are included in light blue. Time-resolved traces are depicted as mean + SD of one representative experiment, measured in duplicate. Summarized data is shown as mean ± SEM of three independent experiments. Scale bar is 20 μm. Source data are provided as a Source Data file.

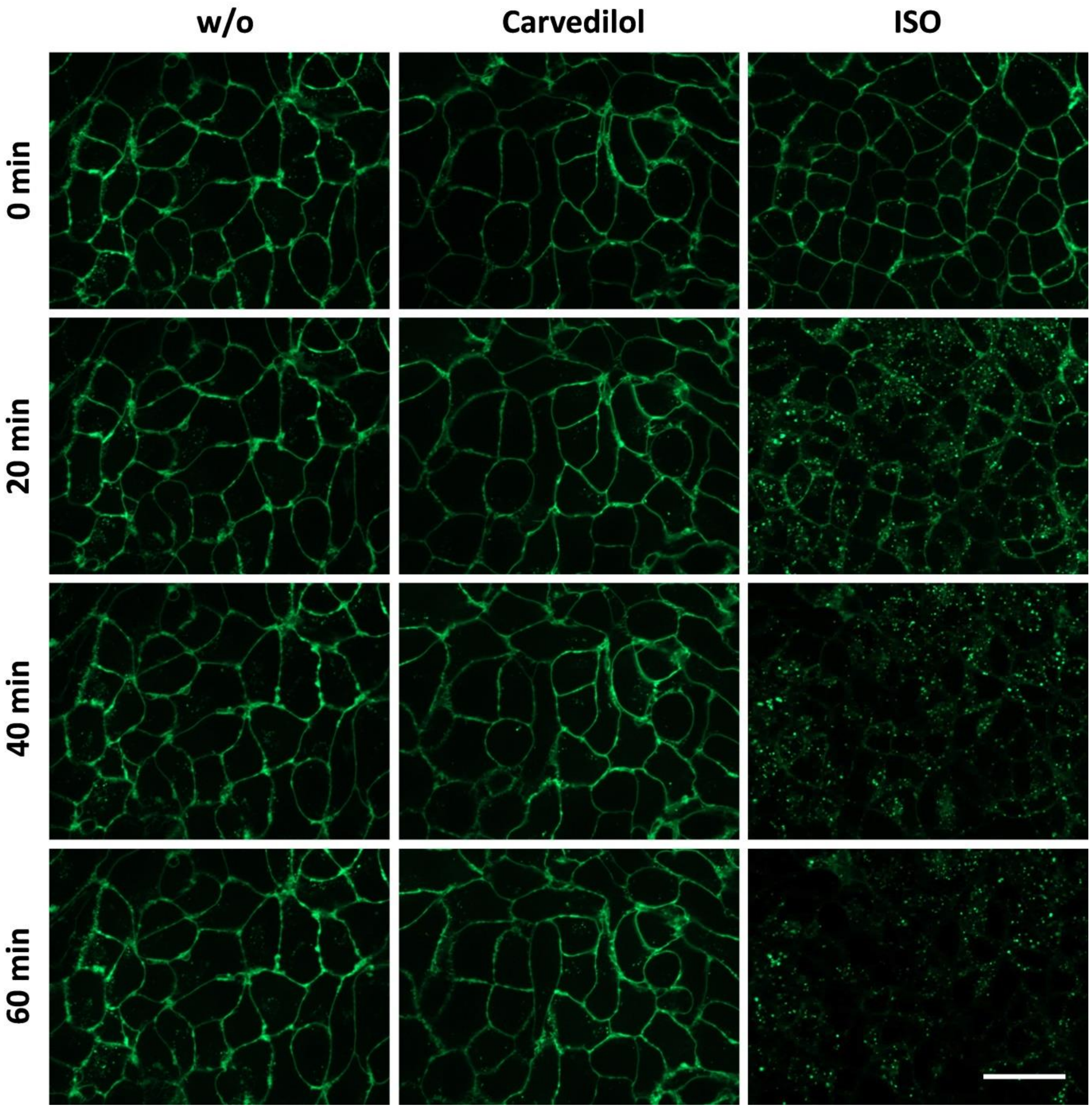

Supplementary Fig. 12: Cellular β$_2$AR redistribution after ligand addition.

Structured-illumination micrographs of wt HEK293 cells stably expressing SNAP-tagged β$_2$ARs treated with buffer (w/o), 10 µM carvedilol or 10 µM ISO for the indicated times using focus control. Scale bar is 20 µm. Representative images from 3 independent experiments.

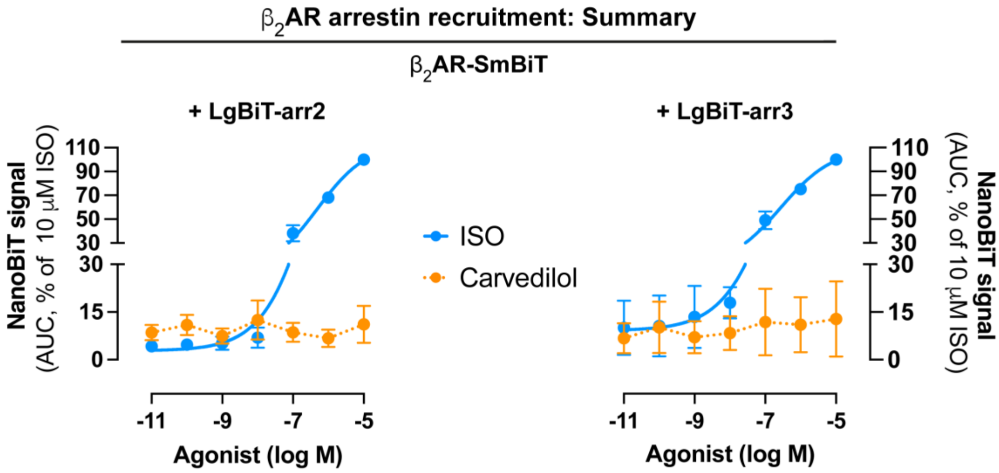

Supplementary Fig. 13: Broken Y-axis view of Fig. 5f.
Summarized data is depicted as mean ± SEM (n = 3). Source data are provided as a Source Data file.

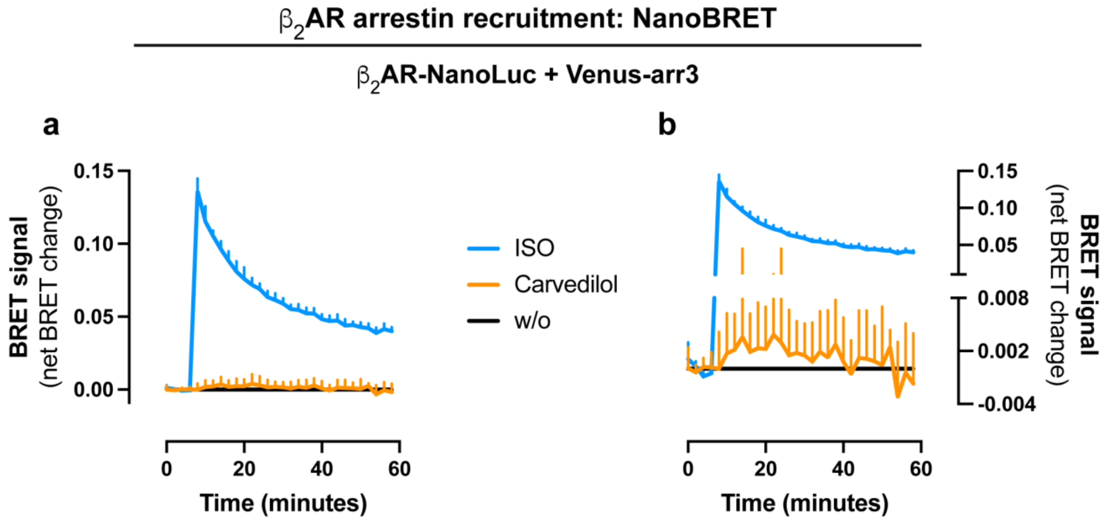

Supplementary Fig. 14: Carvedilol-liganded β_2AR is a poor target for arrestin recruitment. a Ligand-induced BRET between β_2AR-NanoLuc® and Venus-arr3 in wt HEK293 cells after transient transfection. **b** Broken Y-axis view of **(a)**. Summarized kinetic traces of three independent experiments, depicted as mean + SD. Source data are provided as a Source Data file.

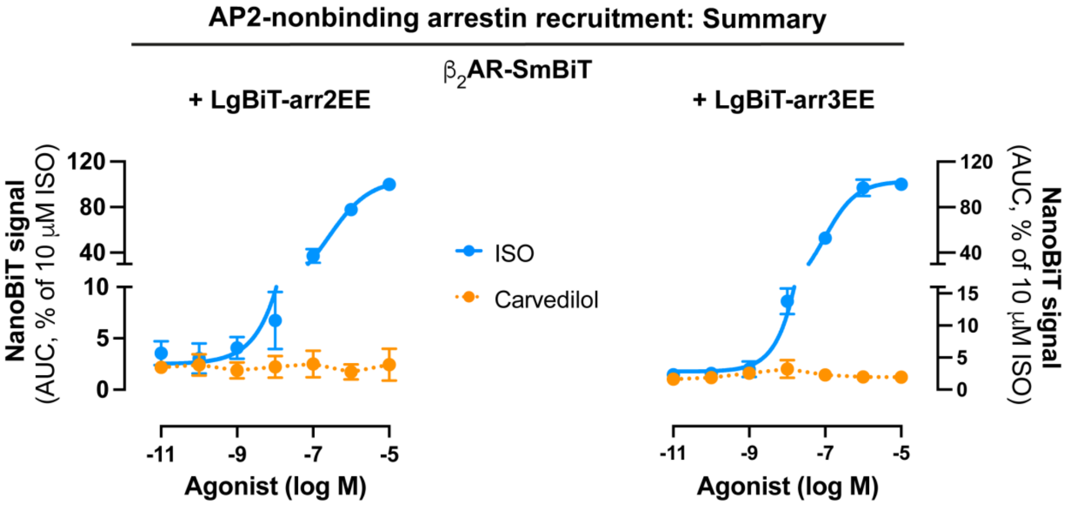

Supplementary Fig. 15: Broken Y-axis view of Fig. 5g.
Summarized data is depicted as mean ± SEM (n = 4). Source data are provided as a Source Data file.

β_2V_2 arrestin recruitment: Summary

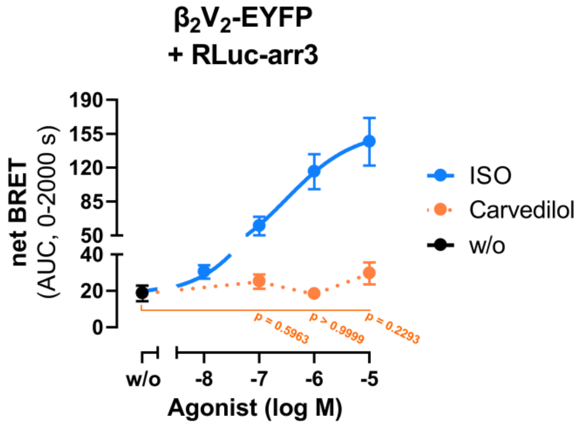

Supplementary Fig. 16: Broken Y-axis view of Fig. 5h.
Summarized data is depicted as mean ± SEM of three independent experiments. Data was analysed by one-way ANOVA with Dunnett´s multiple comparisons post hoc test. Source data are provided as a Source Data file.

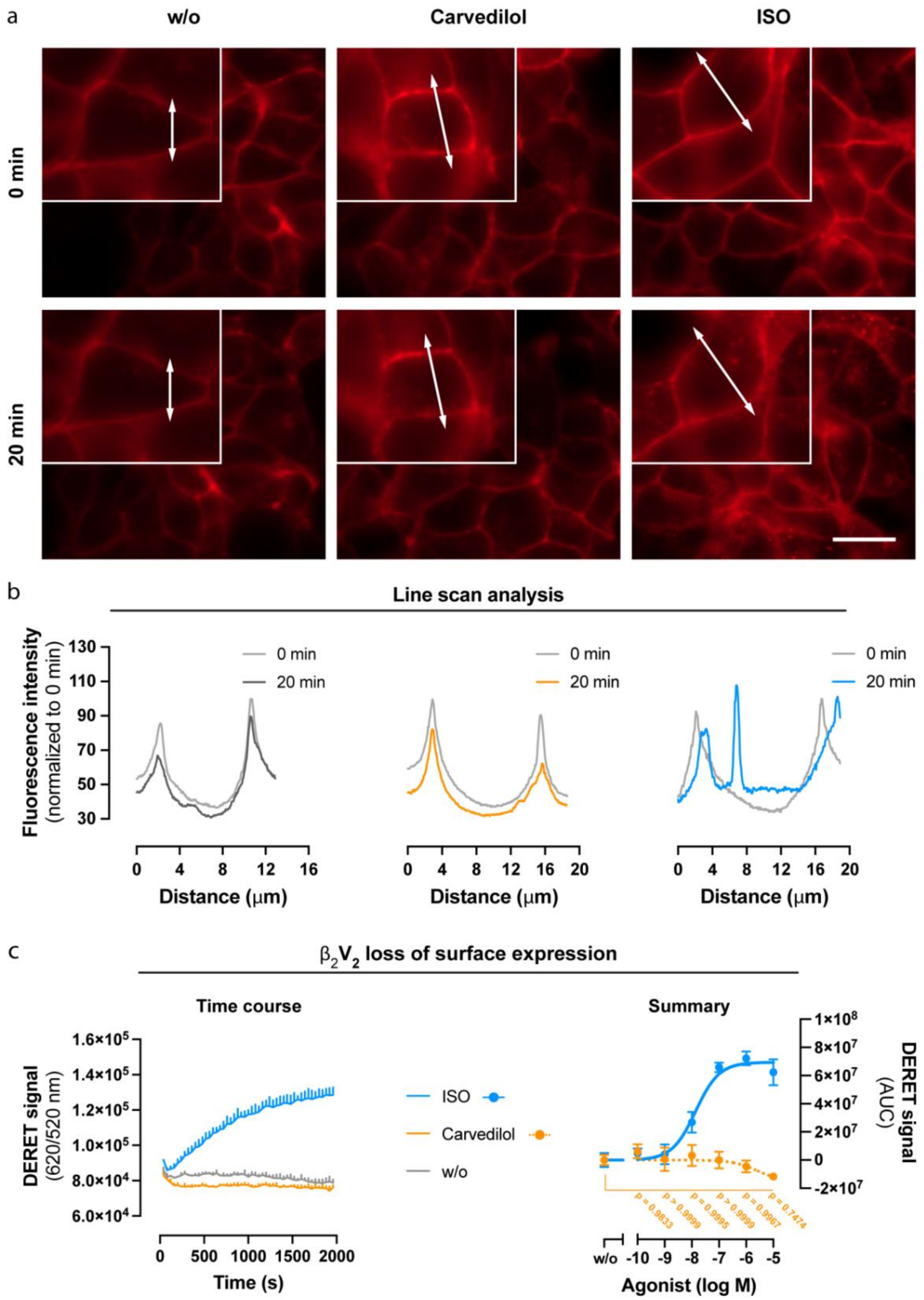

Supplementary Fig. 17: Carvedilol fails to induce internalization of the β_2V_2 chimera. **a** Representative fluorescence micrographs (representative of three independent experiments) of stable SNAP-β_2V_2 wt HEK293 cells before and after addition of either solvent control (w/o), 10 µM carvedilol or 10 µM ISO; scale bar is 20 µm. **b** Line scans corresponding to the panels in **(a)** showing fluorescence intensity distribution after treatment with solvent control, carvedilol and ISO. **c** DERET analysis of SNAP-β_2V_2 surface loss in wt HEK293 cells stably expressing the chimeric receptor after addition of 10 µM carvedilol or 10 µM ISO, and corresponding concentration-effect relationships. Kinetic traces are shown as mean + SD of one representative experiment, measured in duplicate. Summarized data is depicted as mean ± SEM of three independent experiments and was analysed by one-way ANOVA with Dunnett's post hoc test. Source data are provided as a Source Data file.

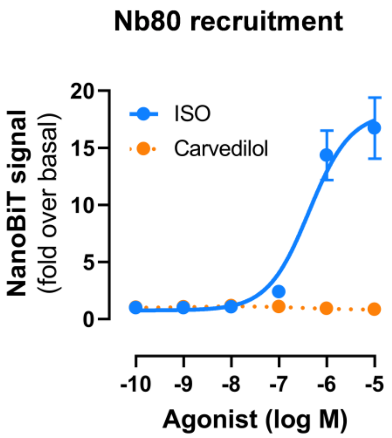

Supplementary Fig. 18: Continuous Y-axis view of Fig. 6a.
Summarized data is depicted as mean ± SEM (n = 5). Source data are provided as a Source Data file.

Supplementary Methods

Supplementary information for metadynamics simulations

Table of contents

1. Supplementary results for metadynamics simulations
2. β_2AR protein treatment and protonation state for MD simulations
3. JSON files for flare plot

1. Supplementary results for metadynamics simulations

We investigated whether the observed differences between ISO and carvedilol can be reproduced at the conformation level in Molecular Dynamics (MD) simulations. Specifically, we conducted multiple walker metadynamics simulations along the A100 activation index. These showed that the free energy surface (FES) of *apo* β_2AR only consists of one minimum at 3.14 A100 units (**Fig. 6b**), while ISO's FES has two minima, one at 1.3 A100 and the second at 51.8 A100 (**Fig. 6b**). This indicates that ISO is able to stabilize an active state, while in the unliganded *apo* form, an active state cannot be stabilized. For reference, the active-state crystal structure of the ternary complex of the β_2AR (PDB ID 3SN6) has an A100 value of 34.4, while the inactive-state structure (PDB ID 2RH1) is at an A100 of -24.1. Carvedilol-bound β_2AR has one minimum at -11.2 A100 and a plateau at around A100 of 18 (**Fig. 6b**).

Upon further evaluation of the secondary structures generated in the simulations, it was noted that in the minima of carvedilol-bound β_2AR, ICL2 was unwound (**Fig. 6c**).This results in less frequent hydrogen bonds formed between $Y141^{ICL2}$ and $D130^{3.49}$ of the DRY motif (a fraction of 0.65 of the frames have at least one hydrogen bond compared to 0.97 in apo and 0.98 in both minima of ISO-bound β_2AR) **(Fig. 6c**). Correct α-helix formation of ICL2 has been described to be important for G_α binding (Dror et al., 2009). Inter-helical hydrogen bonds (occurring in >50% of frames in a minimum) and van der Waals interactions (>80% of frames in a minimum) were calculated with GetContacts (https://getcontacts.github.io/) and results were visualised with flare plots. The flare plots show that carvedilol stabilized a conformation distinct from apo and ISO-bound receptors. Identification of two distinct inactive conformations of the β_2AR reconciles structural and biochemical observations (Dror et al., 2009). We conclude from all the data obtained from simulations that carvedilol stabilizes a different conformation of the β_2AR than ISO. This conformation stabilized by carvedilol is not a fully stabilized active state of the β_2AR, as when ISO is bound, but possibly another conformation that leads to weak partial activity.

Dror, R.O.*, et al.* Identification of two distinct inactive conformations of the beta2-adrenergic receptor reconciles structural and biochemical observations. *Proc Natl Acad Sci U S A* **106**, 4689-4694 (2009).

2. β_2AR protein treatment and protonation state for MD simulations

Histidine protonation (HSD: proton in the δ-nitrogen of the imidazole ring; HSE: proton on the ε-nitrogen; HSP: protons on both nitrogen atoms):

- HSP22
- HSE93
- HSE172
- HSP178
- HSP241
- HSP269
- HSD296

Aspartic/Glutamic acid protonation:

- All charged amino acids are (de)protonated such that the side chain carries a formal charge, unless specify otherwise
- ASP79 protonated (i.e. uncharged) to promote stability of active state
- GLU122 protonated (i.e. uncharged) as surrounded by hydrophobic lipid tails

Post-translational modifications:

- CYS341 palmitoylated

N terminal capped with ACE
C terminal capped with CT3

3. JSON files for flare plot

Interactive flare plots of inter-helical hydrogen bonds (occurring in >50% of frames in a minimum) and van der Waals interactions (>80% of frames in a minimum) from the minima of each simulation can be visualised by uploading the respective JSON files from:

https://doi.org/10.5281/zenodo.7050831 to the following site:

https://gpcrviz.github.io/flareplot/?p=create
