# Supplementary material for: How Carvedilol activates β2-adrenoceptors
Source: Nat Commun. 2022 Nov 19;13:7109. doi: 10.1038/s41467-022-34765-w (PMC9675828; doi:10.1038/s41467-022-34765-w)
Supplement: Supplementary file 3 — Reporting Summary [file 41467_2022_34765_MOESM3_ESM.pdf]

nature portfolio

Corresponding author(s): Evi Kostenis

Last updated by author(s): Nov 3, 2022

Reporting Summary

Nature Portfolio wishes to improve the reproducibility of the work that we publish. This form provides structure for consistency and transparency in reporting. For further information on Nature Portfolio policies, see our Editorial Policies and the Editorial Policy Checklist.

Statistics

For all statistical analyses, confirm that the following items are present in the figure legend, table legend, main text, or Methods section.

n/a	Confirmed	
☐	☒	The exact sample size (*n*) for each experimental group/condition, given as a discrete number and unit of measurement
☐	☒	A statement on whether measurements were taken from distinct samples or whether the same sample was measured repeatedly
☐	☒	The statistical test(s) used AND whether they are one- or two-sided<br>*Only common tests should be described solely by name; describe more complex techniques in the Methods section.*
☒	☐	A description of all covariates tested
☐	☒	A description of any assumptions or corrections, such as tests of normality and adjustment for multiple comparisons
☐	☒	A full description of the statistical parameters including central tendency (e.g. means) or other basic estimates (e.g. regression coefficient) AND variation (e.g. standard deviation) or associated estimates of uncertainty (e.g. confidence intervals)
☐	☒	For null hypothesis testing, the test statistic (e.g. *F*, *t*, *r*) with confidence intervals, effect sizes, degrees of freedom and *P* value noted<br>*Give P values as exact values whenever suitable.*
☒	☐	For Bayesian analysis, information on the choice of priors and Markov chain Monte Carlo settings
☒	☐	For hierarchical and complex designs, identification of the appropriate level for tests and full reporting of outcomes
☒	☐	Estimates of effect sizes (e.g. Cohen's *d*, Pearson's *r*), indicating how they were calculated

Our web collection on statistics for biologists contains articles on many of the points above.

Software and code

Policy information about availability of computer code

Data collection	All data collection has been performed on commercially available software as provided by the manufacturer of the respective device. In particular, MicoWin 2000 (Berthold Technologies GmbH & Co KG, Version: 5.22), Epic Autoalign/Imager Lab View 2009 (Perkin Elmer, Version: 9.0.1f2), PHERAstar FSX reader control (BMG Labtech, Version: 5.41), Zeiss Zen blue edition (Carl Zeiss GmbH, Version: 3.3.89.00000)
Data analysis	Data analysis has been performed on commercially available software. In particular, MARS (BMG Labtech, 3.32), Zeiss Zen blue edition (Carl Zeiss GmbH, Version: 3.3.89.00000). Data and statistical analyses were performed using GraphPad Prism version: 9.1.2, whereas for CardioExcyte96-impedance measurements GraphPad Prism version 8.43 was used. cAMP dynamics assay in adult murine ventricular myocytes was processed in ImageJ/Fiji (https://imagej.net/Fiji).

For manuscripts utilizing custom algorithms or software that are central to the research but not yet described in published literature, software must be made available to editors and reviewers. We strongly encourage code deposition in a community repository (e.g. GitHub). See the Nature Portfolio guidelines for submitting code & software for further information.

Data

Policy information about availability of data

All manuscripts must include a data availability statement. This statement should provide the following information, where applicable:

- Accession codes, unique identifiers, or web links for publicly available datasets
- A description of any restrictions on data availability
- For clinical datasets or third party data, please ensure that the statement adheres to our policy

β2AR 3D structures data used in this study were obtained from the G protein-coupled receptor database (https://gpcrdb.org/structure/3SN6 and https://gpcrdb.org/structure/2RH1). Metadynamics simulations data have been deposited in the open repository Zenodo (https://doi.org/10.5281/zenodo.7050831). All data generated and analyzed during this study are included in this published article and the Supplementary Information. Source data are provided with this paper. Additional data related to this paper may be requested from the authors.

Human research participants

Policy information about studies involving human research participants and Sex and Gender in Research.

Reporting on sex and gender	No human research participants were included in this study.
Population characteristics	n/a
Recruitment	n/a
Ethics oversight	n/a

Note that full information on the approval of the study protocol must also be provided in the manuscript.

Field-specific reporting

Please select the one below that is the best fit for your research. If you are not sure, read the appropriate sections before making your selection.

☒ Life sciences ☐ Behavioural & social sciences ☐ Ecological, evolutionary & environmental sciences

For a reference copy of the document with all sections, see nature.com/documents/nr-reporting-summary-flat.pdf

Life sciences study design

All studies must disclose on these points even when the disclosure is negative.

Sample size	Sample size was chosen based on prior experience of the investigators with similar experiments previously published. The authors have published numerous peer-reviewed papers demonstrating clear positive findings with similar sample sizes for the types of experiments included (PMID: 29362459, 22929080, 29211031, 24275181, 34441183, 35087057, 32561830).
Data exclusions	For analyses of beating frequency changes upon agonist treatment, myocytes with a basal frequency of >60 bpm were excluded. For all other experiments, no data were excluded.
Replication	All experimental findings were reproduced in several independent experiments, as indicated in the figure legends.
Randomization	Randomization was not relevant to our study as it was based on cellular experiments and aimed to test the effect of different agonist on the same sample. Our study was not a clinical trial that is dependent of randomization. Experiments were performed using cells and all variables could be controlled.
Blinding	Blinding was not relevant because there were no subject differences for the different treatments as well as transfection of the cells was conducted by the same investigators.

Reporting for specific materials, systems and methods

We require information from authors about some types of materials, experimental systems and methods used in many studies. Here, indicate whether each material, system or method listed is relevant to your study. If you are not sure if a list item applies to your research, read the appropriate section before selecting a response.

Materials & experimental systems

n/a	Involved in the study
☐	☒ Antibodies
☐	☒ Eukaryotic cell lines
☒	☐ Palaeontology and archaeology
☐	☒ Animals and other organisms
☒	☐ Clinical data
☒	☐ Dual use research of concern

Methods

n/a	Involved in the study
☒	☐ ChIP-seq
☒	☐ Flow cytometry
☒	☐ MRI-based neuroimaging

Antibodies

Antibodies used	All antibodies used are described in the Methods section.<br>Phosphosite specific antibodies were from 7TM antibodies (pS355/pS356-β2AR, Cat. no.: 7TM0029A, dilution 1:200, and pT360/pS364-β2AR, Cat. no.: 7TM0029B, dilution 1:200) and Invitrogen (pSer261-β2AR, Cat. no.: PA5-12977, dilution 1:200).<br>Anti-HA antibody was from Cell Signaling Technology, Cat. no.: 3724, dilution 1:1000.<br>Anti-SNAP-tag antibody was obtained from New England Biolabs, Cat. no.: P9310S, dilution 1:1000.
Validation	Validation for the species (human) and the application (Western Blot) was performed by the manufacturer using fluorescence microscopy and Western Blot analysis. For details, please visit the manufacturers´ web sites.

Eukaryotic cell lines

Policy information about cell lines and Sex and Gender in Research

Cell line source(s)	HEK293 cells and CHO-K1 cells (source: ATCC).<br>ΔQ-GRK HEK293 cells (source: Carsten Hoffmann); Δarr2/3 HEK293 cells and Δsix HEK293 cells (source: Asuka Inoue); SNAP-β2AR HEK293 cell lines and SNAP-β2V2 HEK293 cell line (source: Evi Kostenis). Details and citations on cell generation and characterization are given in the Methods section.
Authentication	Cells were not further authenticated by the authors
Mycoplasma contamination	All cell lines were routinely screened for possible mycoplasma contamination. Results were always negative.
Commonly misidentified lines<br>(See ICLAC register)	No commonly misidentified cell lines were used in the study,

Animals and other research organisms

Policy information about studies involving animals; ARRIVE guidelines recommended for reporting animal research, and Sex and Gender in Research

Laboratory animals	Neonatal mice (CD1 background); 8 - 10 weeks old pmEpac1-camps transgenic mice were previously published (65). Housing conditions: mice were kept in 12/12 h dark/light cycles with food and water ad libido at room temperature (20°C - 24°C) and standard humidity (45% - 60 %).
Wild animals	No wild animals were used in this study.
Reporting on sex	Sex was not considered in the study
Field-collected samples	No field collected samples were used in this study
Ethics oversight	All animal experiments were performed according to institutional and governmental guidelines and approved by the national state authority BGV Hamburg (approval No. ORG_1010). Special approval for harvesting neonatal cardiomyocytes was not required.

Note that full information on the approval of the study protocol must also be provided in the manuscript.
